# Identifying Novel Data-Driven Dietary Patterns via Dimensionality Reduction and Associations with Socioeconomic Profile and Health Outcomes in Ireland

**DOI:** 10.3390/nu15143256

**Published:** 2023-07-23

**Authors:** Daniel T. Burke, Annemarie E. Bennett, Paul Hynds, Anushree Priyadarshini

**Affiliations:** 1 Environmental Sustainability & Health Institute, Technological University Dublin, D07 EWV4 Dublin, Ireland; 2 School of Medicine, Trinity College Dublin, D02 R590 Dublin, Ireland

**Keywords:** dietary patterns, principal component analysis, noncommunicable diseases, BMI, Ireland

## Abstract

Dietary patterns and body mass index (BMI) play a significant role in the development of noncommunicable diseases (NCDs), which are the leading cause of mortality worldwide, including Ireland. A cross-sectional survey was conducted across Ireland to collate respondents’ socioeconomic profiles, health status, and dietary patterns with a representative sample size of 957 adult respondents. Principal component analysis (PCA) and statistical analyses were subsequently employed. To the author’s knowledge, this is the first study to use recent (2021) nationally representative data to characterise dietary patterns in Ireland via dimensionality reduction. Five distinct dietary patterns (“meat-focused”, “dairy/ovo-focused”, “vegetable-focused”, “seafood-focused”, and “potato-focused”) were identified and statistically characterised. The “potato-focused” group exhibited the highest mean BMI (26.88 kg/m^2^), while the “vegetable-focused” group had the lowest (24.68 kg/m^2^). “Vegetable-focused” respondents were more likely to be associated with a categorically healthy BMI (OR = 1.90) and urban residency (OR = 2.03). Conversely, “meat-focused” respondents were more likely to have obesity (OR = 1.46) and rural residency (OR = 1.72) along with the “potato-focused” group (OR = 2.15). Results show that data-derived dietary patterns may better predict health outcomes than self-reported dietary patterns, and transitioning to diets focusing on vegetables, seafood, and lower meat consumption may improve health.

## 1. Introduction

Dietary patterns play a significant role in the development of noncommunicable diseases (NCDs), which are the leading cause of mortality globally [1,2]. Diet-related NCDs, including obesity, cardiovascular diseases (arterial hypertension, myocardial infarction, stroke), diabetes mellitus, some cancers, and osteoporosis, have become more prominent than disease conditions resulting from nutrient deficiencies [3,4]. Global and European rates of all-cause mortality attributed to NCDs are 74% and 90%, respectively [5]. Body mass index (BMI) represents a major predictor for development of diet-related NCDs (e.g., cardiovascular disease and diabetes) and, more recently, COVID-19 severity [6,7,8,9,10]. For example, severe obesity (BMI ≥ 40) was identified as a substantial risk factor for COVID-19-related intensive care unit (ICU) admission and death in the Republic of Ireland (ROI) due to CVOID-19 [8].

Obesity is a prevalent issue in the ROI, with the Healthy Ireland Survey (2022) recently reporting that 35% of the adult population has overweight, while 21% has obesity [11]. The Health Service Executive (HSE) of the ROI reported in their Obesity Policy and Action Plan 2016–2025 that the predictors of obesity include access to healthy and affordable food, cultural and societal norms, education and skill levels, and lifestyle choices [12]. The World Health Organisation (WHO) forecasts that by 2030, 47% of both males and females will have obesity in the ROI, thus placing additional needs on existing health systems [9,13].

A long-term “whole diet” approach examining dietary patterns at the subpopulation level over weeks, months, or years is necessary for identifying associations between dietary patterns and NCDs as chronic disease risks are typically caused by chronic exposures [14,15,16]. Traditional nutritional epidemiology typically examines diet–disease associations on a single nutrient, food product, or food group (e.g., meat, dairy, etc.). However, as foods are consumed in various combinations characterised by synergistic or antagonistic effects, the “whole diet” approach has gained more attention in recent years [14,16,17,18,19,20]. 

As dietary patterns change, there is a need to update current knowledge of food consumption patterns within specific populations to help attenuate prevalent NCDs. In the ROI, four adult-focused food consumption surveys have been conducted since the mid-1990s: the North South Ireland Food Consumption Survey (NSIFCS) (1997–1999; 1379 participants), the National Adult Nutrition Survey (NANS) (2008–2010; 1500 participants), the Survey on Lifestyle and Attitude to Nutrition (SLÁN) (1998; 6539 participants, 2002; 5992 participants, 2007; 10,364 participants), and Healthy Ireland (annual survey) from the Irish Department of Health. These represent the current evidence base for policy recommendations within the ROI, such as developing a “recommended diet” for older adults to maintain optimal health [21,22,23]. Additionally, several studies have shown that socio-economic profiles are known to be associated with dietary patterns among Irish adolescents [24,25,26]. However, over the past decade, there has been relatively little research conducted in the context of the adult Irish population (≥18 years). 

European and Irish dietary patterns have changed in recent decades due to changing demographics, rising incomes, and increasing food supply [27]. Since the 1990s and 2000s, Irish socioeconomic profiles have shifted substantially, significantly contributing to broad-brush dietary changes. Ireland’s multifaceted socioeconomic and demographic changes during the late 1980s included new forms of governance, economic and social growth, improved education, and favourable international investment, including significant investment from the European Union [28]. Alongside these changes, migration into Ireland increased in the 2000s, with the Central Statistics Office (CSO) reporting that the fastest growing ethnic group since 2011 was “Other including mixed background”, with a growth of 14.7% between 2011 and 2016 [29,30]. The ROI has become more ethnically diverse with 17.3% of the total current population born outside Ireland, representing 180 countries [29,30]. The urban–rural divide in the ROI is a significant factor in Irish socio-demographics, with the proportion of people living in rural Ireland equating to 37.3%, significantly higher than the current European Union mean of 27.3% [31]. Accordingly, there is a need to examine contemporary Irish dietary patterns and the accompanying socioeconomic and health associations as these relationships influence dietary patterns and preferences [17,32,33,34].

The rate of obesity in the ROI is expected to rise to 47% by 2030; however, the contemporary relationship between dietary patterns and socioeconomic profile is not well understood [13]. Accordingly, the present study aimed to (i) identify data-driven dietary patterns in the ROI based on food frequency questionnaires (FFQ) and a dimensionality reduction approach, (ii) examine associations between respondents’ socioeconomic profile and attributed dietary pattern, and (iii) assess how dietary patterns and socioeconomic conditions are associated with health outcomes (BMI, diabetes, cardiovascular disease, and stroke). 

These findings will contribute to an improved understanding of the sociodemographic determinants of dietary patterns and the interrelationship with health, thus permitting increasingly evidence-based prevention of NCDs by not focussing on specific nutrients or individual foods but rather on whole-scale dietary change. Appropriate dietary patterns can be recommended through increasingly focused interventions and public engagement. 

## 2. Methodology 

This study was reviewed and approved by the Research Ethics and Integrity Committee of Technological University Dublin (Ref REC-20-85, dated 18 February 2021).

### 2.1. Survey Compilation

Study data were derived from a cross-sectional survey conducted across the ROI, (total population of 5.1 million people [35]) with data collected via a self-administered online questionnaire. Convenience sampling was used to obtain a statistically representative sample size of 957 with a required sample size of 770 (385 in both urban and rural regions), calculated to achieve a confidence interval (CI) of 95% and a margin of error ≤ 5%. Maintaining a representative sample was essential for the survey’s duration, with the mode of dissemination tailored to specific subgroups if and when required based on census results [36]. To ensure the survey was able to capture a representative sample (i.e., demographic cross-section), data were assessed as the survey was administered throughout the ROI, and adjustments were made to the target population (i.e., gender ratio, age range, education level, settlement pattern). For example, if and when it was noted that younger adults were underrepresented, a specific focus was placed on eliciting responses from third-level educational institutions. In the current study, dietary patterns and diets were defined as “the quantities, proportions, variety or combinations of different foods and beverages in diets, and the frequency with which they are habitually consumed” [37]. Survey participants were required to be ≥18 years of age. Urban and rural residency was attributed via application of the self-reported distance between respondents’ place of residence and the closest food item retailer based on the CSO’s report, Measuring Distance to Everyday Services in Ireland [38]. Urban/peri-urban residents were defined as living within 4 km of a food retailer, with all other respondents being defined as rural residents [38]. The survey was distributed across all 26 administrative counties in the ROI.

### 2.2. Questionnaire Design

A comprehensive questionnaire was designed to answer the primary research question:

“What are the common dietary patterns in Ireland, and how do they relate to socio-economic profiles and health outcomes?” 

The questionnaire comprised 62 questions (including all filtering questions) distributed across five subsections: (i) sociodemographic profile, 14 questions; (ii) personal health, 5 questions; (iii) dietary habits, 30 questions; (iv) consumer behaviour, 11 questions; and (v) environmental knowledge, 2 questions (Appendix A Table A1 and Table A2). For the purposes of the current article, target response sets from Survey Section 1, Section 2 and Section 3 have been included for analyses.

The first section of the questionnaire addressed the individual-level demographic and socioeconomic status of the respondent and their household (Appendix A Table A1). These questions were posed in multiple-choice format and formulated based on the pre-existing Irish Census. The respondent’s settlement pattern was determined by asking which county they reside in, how far the nearest food retailer is, if they reside within in walking distance of the nearest public house (bar), restaurant, or café, and how long it would take the respondent to travel to the nearest food retailer via car, public transport, or on foot. Respondents were further asked to self-report their current dietary pattern (the dietary term and associated descriptions were provided to avoid confusion, for example, “I eat meat, fish, and vegetables (omnivorous)”. Based on the respondents’ self-reported dietary pattern, they were asked a series of questions related to the selected dietary pattern. 

The second section of the questionnaire examined the respondent’s self-reported health metrics (i.e., weight, height, and calculated BMI) and health background through multiple choice questions (Appendix A Table A2). BMI was calculated based on self-reported height and weight (weight in kilogrammes divided by the square of the person’s height in metres (kg/m^2^)). BMI was classified according to WHO recommendations as follows: “underweight” (BMI < 18.5 kg/m^2^), “healthy” (BMI 18.5–24.9 kg/m^2^), “overweight” (BMI 25.0–29.9 kg/m^2^), and “obese” (BMI > 30.0 kg/m^2^) [39]. Respondents were also asked if they had been previously diagnosed or currently have a potentially dietary-related health complication including hypertension, diabetes, and coronary heart disease [1,40,41].

The third section of the questionnaire investigated the food consumption habits of the respondent via a semi-quantitative FFQ using 12 comprehensive food groups (Appendix A Table A2). The format and content of dietary questions were based on excerpts of validated surveys: the SLÁN (2009) and NANS (2011) studies [42,43,44]. The FFQ semi-quantitatively assessed consumption of food and beverage servings during the previous 30-day (1-month) period. 

Prior to general survey distribution, the validity, brevity, and clarity of the questionnaire was iteratively tested through a pilot study with 30 respondents, and necessary changes made; pilot response sets were not included for the final analyses.

### 2.3. Survey Completion

The survey was distributed electronically during a six-month period from early July to late December 2021 to account for seasonal variation. SurveyMonkey and Typeform were used to host the survey. The survey was disseminated across the ROI through institutional, public, alumni, and private social networks (Twitter, Facebook, LinkedIn, and Instagram); a nationally broadcast radio show; and institutional mailing lists. Upon clicking the survey link, respondents received the study information leaflet and a notice that by starting the survey, they were providing informed consent to participate. Participants could exit the survey at any time. The survey took an estimated ten minutes to complete.

### 2.4. Statistical Analysis

Descriptive statistics (i.e., central tendency, spread, outlier identification, and frequencies) were obtained for all variables. Means and standard deviations or medians and interquartile ranges were employed to detect outliers among continuous parameters (i.e., self-reported anthropometrics). For nonnormally distributed variables, nonparametric statistical tests were employed. 

Chi-square tests were used to assess bivariate proportional associations between categorical variables, followed by post-hoc testing via standardised residuals. Additionally, dummy variables, odds ratio (OR) estimates, and post-hoc testing using adjusted standardised residuals above and below the threshold of 1.50 were used to determine the presence and magnitude of associations between categorical (dichotomous/nominal) variables [45]. Kruskal–Wallis tests were used to investigate relationships between continuous and categorical variables, followed by post-hoc pairwise comparisons, while Spearman’s Rho was used to assess nonparametric associations between continuous variables. The data were analysed using IBM SPSS Statistics (Version: 28.0.0.0), with statistical significance set at 5% (α = 0.05) by convention. All presented bivariate analyses employed a CI of 95%. 

Principal component analysis (PCA) was utilised for dimensionality reduction to identify distinct data-driven dietary habits within the surveyed population based on self-reported food frequency consumption. To identify food groups for inclusion in PCA, relationships between self-reported consumption from the FFQ and the self-reported dietary pattern were analysed using chi-square tests. PCA was undertaken using Varimax rotation with Kaiser Normalisation to assist in component development and generate factor loading [46]. A nonparametric Kruskal–Wallis one-way ANOVA was used to identify significantly different food groups between self-reported dietary patterns and median consumption frequencies across all fifteen food groups included in the questionnaire. Principal components (PC) with eigenvalues ≥ 0.7 were retained for extraction as primary dietary factors [47]. Retained factors were orthogonally rotated using the varimax method for ease of interpretation [48,49]. Bartlett’s test of sphericity and the Kaiser–Meyer–Olkin (KMO) measure of sampling adequacy were used to determine the suitability of extracted components [46]. 

Factor loadings from developed PCs representing the correlation between identified components and each variable were used to characterise the resulting dietary patterns [50,51]. Factors were ordered and given provisional labels according to the food groups that loaded highly on each PC. Food groups with a factor loading of ≥±0.25 are particularly important in characterising identified dietary patterns, as they indicate a strong association with the identified component [52,53]. Each survey respondent was assigned to one of the extracted PCs based on the individual respondent’s self-reported food frequency consumption and factor loadings [54]. Subsequently, bivariate statistical tests were used to identify relationships between the dietary patterns based on PCA and respondents’ self-reported socioeconomic profiles and personal health. Additionally, multivariate logistic regression was utilised to examine the relationship between self-reported health outcomes and confounding socioeconomic variables (age, household pretaxed income, level of educational attainment, and employment status).

## 3. Results

### 3.1. Characteristics of the Study Population

A total of 1023 respondents initiated the survey between July and December 2021. Once incomplete responses, responses from outside the ROI, and respondents < 18 years of age were removed, 957 respondents remained for analysis (Table 1). 

As shown (Table 1), a higher percentage of respondents were female (57.9%, *n* = 554), with the most frequent age range for both genders being between 25 and 34 years (32.4%, *n* = 310). Mean household size was 3.06 (SD = 1.6) with 16.7% (*n* = 160) of respondents living alone. Approximately one-third (*n* = 266) of respondents reported an annual pre-tax household income in the EUR 25,000–EUR 49,999 range. The calculated median BMI across all respondents was 25.89 kg/m^2^ with no significant difference between gender (*p* = 0.897), with a male and female median BMI of 25.99 and 25.73 kg/m^2^, respectively. When delineated by BMI classification, there were significant differences between gender and BMI (χ^2^(3) = 12.348, *p* = 0.006). Post-hoc analyses of standardised residuals indicated that male respondents were more likely to be overweight (OR = 1.52, 95% CI [1.15, 2.01]) and females were more likely to be obese (OR = 1.49, 95% CI [1.08, 2.02]). Overall, 10.3% (*n* = 99) of respondents self-reported as having or had hypertension, 4.9% (*n* = 47) self-reported having or had diabetes, and 1.8% (*n* = 17) reported having coronary heart disease, with no significant differences based on gender. Respondent age-range and calculated BMI were significantly associated (χ^2^ (5) = 49.536, *p* < 0.001). Calculated BMI medians within the 18–24 age range (23.97 kg/m^2^ range) were significantly lower than the other age ranges, while the 25–34 (25.18 kg/m^2^) age range was significantly lower than the 35–44 age range (26.96 kg/m^2^). 

### 3.2. Data-Driven Dietary Pattern Identification (Principal Component Analyses)

Eight food groups were found to have significant relationships with self-reported dietary patterns (Table 2). 

These eight food groups were included for dimensionality reduction as these provided components that explained significantly greater variance than when all food groups from the FFQ were included (i.e., saturated PCA). Eigenvalues ≥ 0.70 revealed five major dietary patterns and explained 79% of the variance within the survey cohort. The resulting principal components (PC) were supported by a KMO value of 0.683 and Bartlett’s test of sphericity < 0.001. The PCs were labelled “meat-focused (PC1)”, “dairy/ovo-focused (PC2)”, “vegetable-focused (PC3)”, “seafood-focused (PC4)”, or “potato-focused (PC5)” (Table 3). 

The “meat-focused” component exhibited the largest explained variance within the population at 28.7% and was characterised by high positive loadings for both non-red meat and red meat, in addition to positive loadings for dairy, seafood, and potatoes. The only negative loadings in PC1 were found for the nuts and seeds food group. The “vegetable-focused” component (PC3) was characterised by having the highest positive loadings for both vegetables and nuts/seeds. Similarly, the “seafood-focused” component (PC4) was characterised by the highest positive loadings for seafood. Smaller positive loadings within PC4 were observed for red meat, nuts/seeds, and eggs, alongside a negative loading for dairy. Lastly, the “potato-focused” component (PC5) was characterised by having the highest positive loading for potatoes, with slightly positive loadings for red meat and eggs. The food frequency consumption for each food group delineated by PCA-derived dietary patterns is presented in Appendix A (Table A3).

### 3.3. PCA-Derived Dietary Patterns and Self-Reported Diet

**Figure 1:**
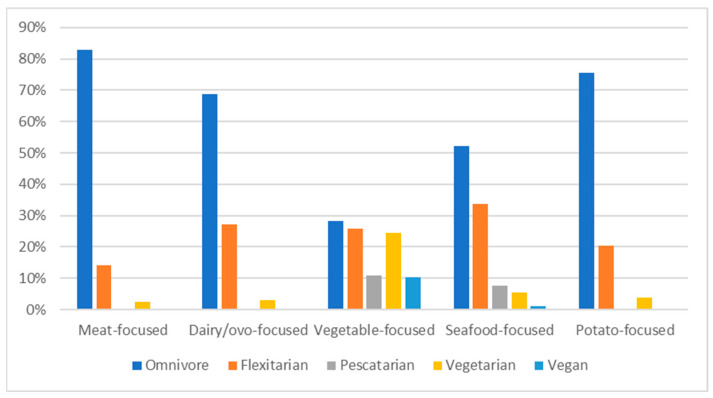
Membership to principal component of dietary patterns according to self-reported dietary pattern. Significant difference between omnivores (*n* = 606), flexitarians (*n* = 218), pescatarians (*n* = 32), vegetarians (*n* = 76), and vegans (*n* = 25) in each PC (chi-squared test; *p* < 0.001).

Significant differences were identified between data-driven dietary patterns (PCs, Figure 1) and respondents’ self-reported dietary pattern (χ^2^ (16) = 299.138, *p* < 0.001) (Figure 1). Omnivores were substantially more likely to be within the “meat-focused” and “potato-focused” groups and unlikely to be in the “vegetable-focused” group. Flexitarians and pescatarians were likely to be in the “seafood-focused” group and not the “meat-focused” group. Pescatarians, vegetarians, and vegans were all likely to be in the “vegetable-focused” group and unlikely to be in the “meat-focused” and “dairy/ovo-focused” groups.

### 3.4. PCA-Derived Dietary Patterns and Socioeconomic Profiles

The socioeconomic and health profiles for each of the five PCA-derived dietary patterns are shown in Table 4. Several statistically significant relationships (sex, ethnicity, settlement pattern, employment status, occupation, household composition, monthly individual food expenses, and diet duration) were identified between the PCA-derived dietary patterns and the respondents’ socioeconomic profiles and health metrics.

Bold values describe the PC with the highest demographic and health characteristics. Table 5 presents calculated adjusted odds ratios (aORs) and CI for the significant associations between socioeconomic profile and attributed PCA-derived dietary pattern. Gender (χ^2^ (4) = 19.571, *p* < 0.001) and ethnicity (χ^2^ (16) = 53.776, *p* < 0.001) were significantly different across the PCA-derived diets. As shown, females were twice as likely to be associated with the “vegetable-focused” diet. Respondents of Irish ethnicity were 3.51 times more likely to follow the “potato-focused” diet while European/non-Irish white respondents were 2.21 times more likely to be associated with the “vegetable-focused” diet and less likely associated with the “meat-focused” (aOR = 0.51, 95% CI [0.32, 0.81]) or “potato-focused” (aOR = 0.18, 95% CI [0.06, 0.57]) diets. 

Respondents of mixed ethnicity were 3.89 times more likely to follow the “seafood-focused” diet. Respondent’s self-reported dietary pattern duration and PCA-derived diets were significantly associated (χ^2^ (16) = 98.591, *p* < 0.001); respondents categorised in the “meat-focused” group were 2.6 times more likely to have followed the same diet for more than fifteen years and respondents in the “potato-focused” dietary group were 1.7 times more likely to have been following the same diet for more than fifteen years. Conversely, “vegetable-focused” respondents were 2.5 times more likely to follow the same diet for one to five years and twice as likely to follow the same diet for six to ten years. The “seafood-focused” diet group was also found to have switched to their current diet relatively recently with this group 2.2 times more likely to have adhered to their current diet for less than a year. PCA-derived and self-reported dietary patterns were found to be not significantly associated with respondents’ self-reported household income and level of educational attainment. 

No overarching statistical relationships were found between PCA-based dietary patterns and age group (χ^2^ (20) = 30.205, *p* = 0.067), educational attainment (χ^2^ (16) = 24.848, *p* = 0.073), or pre-tax household income (χ^2^ (24) = 22.336, *p* = 0.559). However, PCA-based dietary patterns were closer to significance than self-reported diets and age group (χ^2^ (20) = 23.567, *p* = 0.262) and educational attainment (χ^2^ (16) = 17.996, *p* = 0.324). Subsequent post-hoc testing via multivariate logistic regression identified some category-specific (i.e., measurement level) associations. Respondents with postgraduate qualifications were more likely to have a “vegetable-focused” diet (aOR = 2.15, 95% CI [1.19, 3.86]) and less likely to have a “potato-focused” diet (aOR = 0.47, 95% CI [0.23, 0.96]). Similarly, respondents with a doctorate degree were more likely to be “vegetable-focused” (aOR = 3.40, 95% CI [1.48, 7.77]) and less likely to be “potato-focused” (aOR = 0.20, 95% CI [0.04, 0.97]).

Further examination revealed a statistically significant relationship between the level of educational attainment and PCA-based dietary patterns (χ^2^ (8) = 19.424, *p* = 0.013) when respondents were classified into broader educational groups (i.e., up to and including secondary school, undergraduate degree, and postgraduate qualification), but not for self-reported dietary patterns (χ^2^ (8) = 5.119, *p* = 0.745). As shown in Table 5, respondents with a postgraduate qualification were less likely to follow the “meat-focused” (aOR = 0.75, 95% CI [0.57, 0.996]) and “potato-focused” diet (aOR = 0.54, 95% CI [0.34, 0.84]), but more likely to be grouped in the “vegetable-focused” diet group (aOR = 1.56, 95% CI [1.15, 2.12]). Conversely, respondents with an educational attainment level up to and including secondary school were more likely to have a “potato-focused” diet (aOR = 1.75, 95% CI [1.15, 2.67]) and less likely to be in the “vegetable-focused” diet group (aOR = 0.67, 95% CI [0.47, 0.96]).

**Figure 2:**
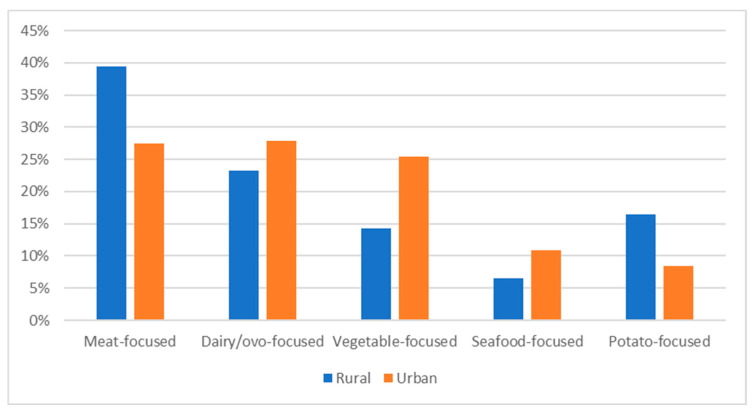
Membership to principal component (PC) of dietary patterns according to settlement pattern. Significant difference between urban (*n* = 678) and rural (*n* = 279) in each PC (chi-squared test; *p* < 0.001).

Settlement pattern was significantly associated with PCA-derived dietary patterns (χ^2^ (4) = 37.698, *p* < 0.001) (Figure 2); rural respondents were 2.15 and 1.72 times more likely to be associated with “potato-focused” and “meat-focused” diets, respectively. Conversely, respondents residing in urban areas were twice as likely (aOR = 2.03, [1.39, 2.96]) to be associated with a “vegetable-focused” diet.

Employment status (χ^2^ (32) = 49.947, *p* = 0.023) and occupation (χ^2^ (32) = 51.239, *p* = 0.017) were both significantly associated with PCA-derived dietary patterns. Respondents working in the “Engineering, architecture, manufacturing, building, construction” field were twice as likely to be categorised in the “meat-focused” and “seafood-focused” diet groups, while respondents working in “education” were 2.1 times more likely to be categorised in the “vegetable-focused” diet group. Total household size (F(4) = 14.820, *p* = 0.005) and living with or without children (<18 years) (χ^2^ (4) = 18.886, *p* < 0.001) were significantly associated with PCA-derived diets. “Seafood-focused” diet respondents were associated with a smaller household than both the “dairy/ovo-focused” and “potato-focused” diet groups. Respondents living without children were 1.5 times more likely to follow a “vegetable-focused” diet, while respondents living with children were 1.9 times more likely to follow a “potato-focused” diet. 

### 3.5. Associations between Self-Reported and PCA-Derived Dietary Patterns and Self-Reported Health

#### 3.5.1. Body Mass Index

**Figure 3:**
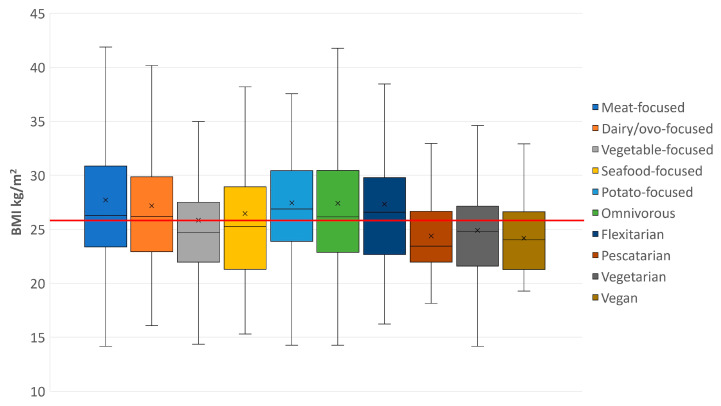
Box and whisker chart for each PCA-derived and self-reported diet with the global median (25.89 kg/m^2^) shown as a horizontal red line.

Self-reported flexitarians exhibited the highest BMI of 26.58 kg/m^2^, while self-reported pescatarians had the lowest median BMI of 23.43 kg/m^2^ (Table 6). The “potato-focused” diet had the highest reported median BMI of 26.88 kg/m^2^, whereas the “vegetable-focused” diet had the lowest at 24.68 kg/m^2^. A significant association was identified between calculated BMI and self-reported diet (F(4) 19.778, *p* < 0.001) with post-hoc tests identifying self-reported omnivores and flexitarians as having a significantly higher BMI than vegetarians. As shown (Figure 3), self-reported omnivores and flexitarians had a higher BMI higher than the sample median of 25.89 kg/m^2^. Significant associations were also found between respondents attributed dietary pattern and calculated BMI (F(4) = 19.008, *p* < 0.001); the median BMI (24.68 kg/m^2^) of the “vegetable-focused” diet was significantly lower than the median BMI (26.88 kg/m^2^) of the “potato-focused” and “meat-focused” (26.26 kg/m^2^) dietary patterns. 

**Figure 4:**
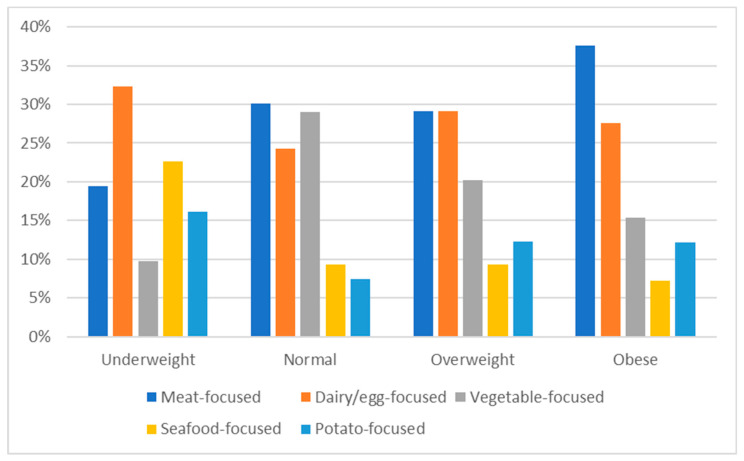
Membership of BMI weight class delineated by PCA-derived diet. Significant difference between underweight (*n* = 31), healthy (*n* = 366), overweight (*n* = 302), and obese (*n* = 221) for each PC dietary pattern (chi-squared test *p* < 0.001).

A significant association was identified between self-reported dietary patterns (χ^2^ (12) = 28.457, *p* = 0.005), PCA-derived dietary patterns (χ^2^ (12) = 34.373, *p* < 0.001), and BMI classification with post-hoc analysis revealing that self-reported omnivores were 1.7 times more likely to have obesity (Figure 4). Respondents attributed to the data derived “seafood-focused” diet were three times more likely to be underweight. “Vegetable-focused” respondents were 1.9 times more likely to have a healthy BMI and less likely to have obesity (OR = 0.57), while respondents consuming a “meat-focused” diet were 1.46 times more likely to have obesity (Table 7).

Urban respondents exhibited a significantly (χ^2^ (1) = 5.672, *p* = 0.017) lower median BMI (25.66 kg/m^2^) than rural respondents (26.54 kg/m^2^). Employment status (χ^2^ (8) = 24.243, *p* = 0.002) and occupation (χ^2^ (8) = 26.020, *p* = 0.003) were also significantly associated with BMI; unemployed respondents and students (with and without parttime jobs) exhibited a lower median BMI than the population median of 25.89 kg/m^2^. For example, the median calculated BMI for respondents working for payment or profit (26.13 kg/m^2^) was significantly higher than students without a parttime job (23.72 kg/m^2^). Respondents working in “computing, IT, scientific and technical” fields had a significantly lower median BMI (23.94 kg/m^2^) than respondents working in “services” (28.02 kg/m^2^) and “healthcare” (26.12 kg/m^2^). Respondents living with household members < 18 years exhibited a higher BMI than the population median of 25.89 kg/m^2^ (χ^2^ (1) = 4.234, *p* = 0.040). 

#### 3.5.2. Self-Reported Health Conditions

There were no significant associations between self-reported dietary pattern and current/previous incidence of hypertension (χ^2^ (4) = 4.467, *p* = 0.347), diabetes (χ^2^ (4) = 0.945, *p* = 0.918), or coronary heart disease (χ^2^ (4) = 5.887, *p* = 0.208). Conversely, for PCA-derived dietary patterns, there was a significant association (χ^2^ (4) = 15.612, *p* = 0.004) between coronary heart disease and the “seafood-focused” diet, with this group 5.4 times more likely to report having coronary heart disease. While no significant relationships were found between PCA-derived diets, hypertension (χ^2^ (4) = 7.199, *p* = 0.126), and diabetes (χ^2^ (4) = 2.427, *p* = 0.658), these exhibited lower *p* values than the self-reported diets. 

#### 3.5.3. Associations between Self-Reported Health and Socioeconomic Profile

Pretaxed household income was significantly associated with the incidence of hypertension (*p* = 0.02). Respondents with a pretaxed annual household income between EUR 25,000 and EUR 49,999 (aOR = 0.41, 95% CI [0.20, 0.86]), EUR 75,000 and EUR 99,999 (aOR = 0.34, 95% CI [0.14, 0.83]), and EUR 100,000 and EUR 124,999 (aOR = 0.28, 95% CI [0.09, 0.81]) were less likely to have or had hypertension. The incidence of coronary heart disease was significantly associated with unemployment (*p* = 0.01), as unemployed respondents were more likely to have reported coronary heart disease (aOR = 10.74, 95% CI [1.65, 69.98]). 

## 4. Discussion

The present study successfully employed PCA to identify five distinct dietary patterns among 957 adult respondents in the ROI and identified associations with self-reported health outcomes and socioeconomic variables. The five PCA-derived dietary patterns were “meat-focused”, “dairy/ovo-focused”, “vegetable-focused”, “seafood-focused”, and “potato-focused”. The Healthy Ireland Survey 2022 found that 2% of the population are underweight, 41% have a healthy BMI, 35% are overweight, and 21% have an obese BMI [11]. These results are relatively similar to findings from the current study, with 3.4% of respondents being underweight, 39.8% having a healthy BMI, 32.8% being overweight, and 24% being obese, which speaks to the representativeness of the findings. Settlement patterns reported in this study were also comparable with the results from the CSO report, *Urban and Rural Life in Ireland 2019*, reporting 31.4% of people live in rural areas, while the current study included 29.2% of respondents residing in rural areas [31]. The *2021 Dietary Lifestyle Report* found that the percentage of people in the ROI adhering to a vegan diet was 2%, 9% for vegetarians, and 19% for flexitarians [55]. Similarly, the results of the current study found that 2.6% of the respondents self-identified as vegan, 7.9% as vegetarian, and 22.8% as flexitarians. 

Two previous studies by Hearty et al. (2009, 2013) used PCA and cluster analysis to examine existing dietary data collected from the ROI (North/South Ireland Food Consumption Survey 1997–1999 and the National Teens Food Survey 2005–2006) and reported that both PCA and cluster analysis identified similar dietary patterns from the same datasets [50,56]. The study by Hearty et al. (2009) used PCA to identify four dietary patterns among the adult population of Ireland based on 1997–1998 dietary data, namely “unhealthy foods and high alcohol”, “traditional Irish”, “healthy foods”, and “sweet foods & breakfast cereal” [56]. The “traditional Irish” diet was comparable to the “meat-focused” and “potato-focused” diets, as the factor loadings for potatoes and red meat were both high (>0.75). Likewise, the “healthy foods” diet was comparable to the “vegetable-focused” diet with high factor loadings for vegetables (>0.60). While the follow-up study by Hearty et al. (2013) focused on adolescents based on dietary data from 2005–2006, similar dietary patterns were again identified [50]. The adolescent “healthy foods” group was similar to the “vegetable-focused” and “seafood-focused” dietary patterns, while the “traditional Irish” group was comparable to the “meat-focused” and “potato-focused” diet groups.

While relatively similar dietary patterns were identified in both studies by Hearty et al. (2009, 2013), the present study identified unique dietary patterns with a higher resolution/clearer boundary (i.e., explained variance reported for the previous adult and adolescent studies was 28% and 28.5%, respectively, while explained variance in the current study was 79%) [50,56]. The previous studies incorporated more food groups within their PCA (thirty-three food groups in Hearty et al. (2009) and thirty-two in Hearty et al. (2013) [50,56]. Comparatively, this study reduced the number of food groups even further to eight groups and found them to be significantly explanatory with respect to self-reported dietary pattern. 

Socio-demographics in the ROI have changed substantially since previous dietary surveys, likely influencing generated principal components. Prendiville et al. (2021) analysed metabolomic dietary data from the Irish NANS study (2008–2010) via cluster analysis and identified four distinct dietary patterns: “moderately unhealthy”, “convenience”, “moderately healthy”, and “prudent” [57]. Although cluster analysis was used, overlapping dietary patterns were found in relation to the current study. PCA-derived “meat-focused” and “potato-focused” diets were similar to the “moderately unhealthy” and “convenience” diets, as both red and white meat consumption were high. Additionally, the “moderately healthy”, and “prudent” diets align with the PCA-derived “vegetable-focused” diet with frequent consumption of vegetables. Notably, no previous Irish studies have explored socioeconomic characteristics or health as they related to data-driven dietary pattern, nor have they examined self-reported dietary preference (i.e., omnivorous, flexitarian, pescatarian, vegetarian, vegan).

I. Self-reported and data-derived dietary patterns associated with health and socioeconomics

Previous studies [58,59,60] have reported a significant mismatch between self-reported dietary pattern and the food groups being consumed. For example, in the current study, 16% of vegans were found to have reported consuming dairy products “at least once a day” while 4% reported consuming eggs “at least once a day” (Appendix A Table A4). Similarly, self-reported vegetarians reported consuming seafood, red meat, and non-red meat at various frequencies. These discrepancies between actual food consumption and self-reported dietary patterns may contribute to the lack of significance between self-reported dietary patterns and health outcomes (diabetes, coronary heart disease, and hypertension). 

Findings suggest that respondents may have differing definitions of self-perceived dietary patterns compared to those generally recognised as omnivorous, flexitarian, pescatarian, vegetarian, and vegan. Thus, caution should be exercised when interpreting self-reported dietary patterns from an epidemiological perspective. Additionally, previous studies have reported that self-identified vegetarians and vegans tend to have healthier lifestyles, including, for example, healthier food choices, higher levels of physical activity, lower prevalence of smoking, and less risky alcohol consumption, thus potentially confounding epidemiological analyses [58,61,62]. Furthermore, increased proliferation of processed plant-based meat alternatives, refined carbohydrates with high sugar content, highly processed snacks and fast foods, traditional plant-based foods, and whole grains might be replaced and possibly align dietary risk to more “normal” diets [61,63]. Therefore, as respondents might not accurately self-report their current diet, coupled with associations between vegetarianism/veganism and healthy lifestyle choices, PCA-derived dietary patterns (or other “unsupervised” statistical methods) may be a more accurate approach to identifying an individual’s true dietary pattern. 

Results from the current study regarding associations between dietary pattern and BMI were broadly in line with previous dietary studies whereby respondents reporting lower levels of meat consumption were found to have lower BMI [57,64,65,66,67]. Watling et al. (2022), reported that “regular meat eaters” and “low meat eaters” and had a mean BMI of 27.9 kg/m^2^ and 27 kg/m^2^, respectively, similar to this study where self-reported omnivores and flexitarians and PCA-derived “meat-focused”, “dairy/ovo-focused”, and “potato-focused” diet groups all exhibited a mean BMI of 27.17 and 27.70 kg/m^2^. 

Conversely, respondents adhering to diets with lower meat consumption (e.g., pescatarian, vegetarian, vegan, “vegetable-focused”, and “seafood-focused”) exhibited a lower mean BMI. Interestingly, the self-reported diet with the lowest median BMI was the pescatarian diet, potentially due to the small sample size and/or the recent proliferation of increasingly processed and ultra-processed plant-based meat alternatives contributing to increasing BMI within the vegetarian and vegan subgroups [68,69]. Watling et al. (2022) reported similar results from an eleven-year longitudinal study in the United Kingdom, with the pescatarian diet exhibiting a lower mean BMI (25.3 kg/m^2^) than the vegetarian diet (25.7 kg/m^2^). These similarities might be attributed to relatively similar food cultures across the British Isles [70].

Paradis et al. (2009) found that respondents who followed the “Western” diet (high in red meats and potatoes) were more likely to have obesity (OR = 1.82), similar to the omnivorous (OR = 1.76) and “meat-focused” (OR = 1.46) diets. Additionally, respondents adhering to both the “prudent” diet (high in vegetables, eggs, fish, and seafood) reported by Paradis et al. (2009) and the current study’s “vegetable-focused” diet were less likely have obesity ((OR = 0.62) and (OR = 0.57), respectively) [65]. Over the past decade, several high-meat diet strategies have been developed to reduce BMI and improve health (i.e., Paleo, carnivore, ketogenic); however, these diets are predominantly based on personal impressions and reports published in books and magazines rather than on scientific evidence, and findings from this study seem to indicate heavy meat diets are not associated with a healthy BMI [71,72]. Therefore, more research is required into the relationship with high-meat dietary patterns, health, and personal activity levels.

To date, this is the first Irish study to specifically identify a “seafood-focused” diet. This dietary pattern was associated with the smallest PCA-derived subgroup (9.6%). This finding may be attributed to several factors: the ROI is an island, with no location situated further than 100 km from the coast. Moreover, domestic seafood consumption has been estimated to have increased from 7% in 1961 to 16% in 2013 [73,74] and the relatively new emergence of the “seafood-focused” diet group might not have been identified in previous studies (NSIFCS; 1997–1999, and the NANS; 2008–2010).

Additionally, the only significant association between self-reported dietary patterns or PCA-derived diets and health outcomes was that respondents categorised in the “seafood-focused” dietary group were 5.4 times more likely to have coronary heart disease. Upon further examination, the “seafood-focused” diet group was also 2.2 times more likely to have followed their food consumption pattern for less than one year. It is unclear if initiation of this dietary shift was due to from advice from medical professionals, self-research and motivation, or recommended through an acquaintance, thus further research is required to clarify this association. However, when health outcomes and dietary duration are considered together, reverse causality is the most probable explanation for this finding, as respondents who have coronary heart disease transitioned to an increasingly seafood-orientated diet to improve health. Previous meta-analyses of observational studies have reported a positive association between fish intake and decreased risk of stroke, coronary heart disease, and cancer [67,75]. Dale et al. (2019) reported that patients with coronary heart disease who consumed lean or fatty fish had reduced blood pressure compared to those who consumed lean meat [75]. As aging (65+) populations are increasingly susceptible to stroke, coronary heart disease, and cancer, and this subpopulation in the ROI is predicated to increase from 629,800 persons (2016) to nearly 1.6 million by 2051, more “seafood-focused” diets may be prescribed to improve health outcomes [29]; therefore, further examination of the “seafood-focused” diet in an Irish context is required.

Settlement pattern was significantly associated with PCA-derived dietary patterns, with rural respondents more likely classified in the “potato-focused” and “meat-focused” dietary groups, while respondents from urban areas more likely adhered to a “vegetable-focused” diet. These results are similar to the study by Layte et al. (2011), who reported that the distance to the nearest food store was related to dietary quality and socioeconomic status, with individuals residing closer to larger and higher-density food outlets and of higher socioeconomic standing exhibiting a significantly better diet in terms of cardiovascular risk [76,77].

The present study found that respondents’ self-reported household income and level of educational attainment were not significantly associated with PCA-derived or self-reported dietary patterns. However, the relationship between PCA-based diets and age group (χ^2^ (20) = 30.205, *p* = 0.067) and educational attainment (χ^2^ (16) = 24.848, *p* = 0.073) were closer to significance than self-reported diets and age group (χ^2^ (20) = 23.567, *p* = 0.262) and educational attainment (χ^2^ (16) = 17.996, *p* = 0.324). When education levels were reclassified into three categories (up to and including secondary school, undergraduate degree, and postgraduate qualification), a statistically significant relationship was reported with PCA-based diets (χ^2^ (8) = 19.424, *p* = 0.013), and not with self-reported dietary patterns (χ^2^ (8) = 5.295, *p* = 0.745), speaking to the credibility of the PCA-derived dietary patterns. Higher levels of educational attainment were associated more with the “vegetable-focused” diet and less with a “meat-focused” dietary pattern. These results mirror previous studies that found that persons with a higher level of educational attainment consumed less meat and more vegetables [78,79]. In terms of household composition, it was found that households without children were more likely to be in the “vegetable-focused” group. Perhaps, households without children are able to spend more money on vegetables, are older, and are more health conscious. Previous research by Kamphuis et al., 2006 and Lee-Kwan et al., 2017 reported that household income has a positive association with vegetable consumption and households with a lower household income consume less vegetables [80,81]. Moreover, investigation among a nationally representative sample to examine the relationships between socioeconomics (i.e., household size and income), dietary patterns, and certain health outcomes is recommended.

II. Improving dietary patterns

Respondents categorised within the “vegetable-focused” and “seafood-focused” diet groups reported varying degrees of red and non-red meat consumption in the current study. The relatively lower observed levels of meat consumption may also attribute to a lower BMI, and meat consumed in low quantities coupled with more vegetables has been shown to improve health (i.e., lower BMI and lower risk of developing cancers) [67]. There are possibilities to improve the Irish diet; just one in four Irish residents (26%) report that they eat five or more portions of fruit and vegetables daily, with a similar proportion (22%) stating that they do not eat fruit or vegetables every day [12]. However, the Ireland: Country Health Profile 2021 report found that, compared to other countries in the European Union, fruit and vegetable consumption in the ROI was among the highest, which may explain the prevalence of the “vegetable-focused” and “potato-focused” diet in the study cohort [82].

In recent years, the Mediterranean, Atlantic, and Nordic diets have gained attention for their health benefits based on evidence from epidemiologic studies and clinical trials indicating that these dietary patterns are associated with reduced incidence of NCDs ranging from cardiovascular disease to cancer [7,83,84]. Accordingly, the unique food culture of Ireland should be improved by shifting away from heavy meat, dairy, egg, and potato consumption to a contemporary North Atlantic/Hibernian/Eireann/Irish diet focusing on vegetable, seafood, and lower meat consumption. In conjunction with the promotion of a healthier North Atlantic/Hibernian/Eireann/Irish diet, both the WHO and the HSE of the ROI have outlined several effective interventions on diet [12,85]. These include policy initiatives such as taxing unhealthy products, regulating foods high in saturated fats, salt, and sugar, restricting “junk food” advertising, overhauling agricultural subsidies that make certain ingredients cheaper than others, and supporting local food production so that consumers have access to healthy, fresh, and nutritious foods [12]. Likewise, educational interventions have been shown to be effective, particularly when adolescent dietary change is the focus. Previous studies that focused on adolescent dietary patterns and food choice in the ROI found that Irish adolescents are influenced more by external factors, such as the smell and taste of food, the sight of food, or being around others who are eating a certain type of food, i.e., their physical and social environment [26,50]. It has also been suggested that food consumption patterns established early in life show long-term stability throughout life [50]. 

Similarly, older adults and persons of lower socioeconomic status and/or lower levels of educational attainment should be encouraged to improve dietary habits [24,86,87]. Accordingly, changes to the physical environment (more fresh vegetable offerings, school vegetable gardens, reduced volumes of meat and ultra-processed food being served) and social environment (e.g., promoting food education) pertaining to food may prove effective in improving diets that can improve diets throughout life and prevent diet-related NCDs [88,89]. Subpopulations in the ROI, such as male farmers, have been regarded as “hard-to-reach” due to rural settlement patterns and generally lower educational attainment; therefore, the promotion of dietary change should cater to specific groups as there is no one size fits all solution to implementing dietary change [9]. Additionally, since results of this study show a relationship between PCA-derived dietary patterns and levels of educational attainment, public health and environmental campaigns should focus on promoting more vegetable-centric diets to less-educated groups (i.e., secondary education level) to encourage diets with less meat consumption [78]. Regardless, promoting dietary change to healthier diets should lead to a reduction in BMI and a subsequent reduction in NCD occurrence. Health and dietary pattern promotional messaging have been shown to be one of the most effective, low-cost interventions available for addressing dietary change and are highly cost-effective when targeted at younger people [26]. The findings of this research will allow for the improvement of existing policy measures in both the ROI and the European Union. “Healthy Ireland” and “European Green Deal—Farm to fork strategy” are programmes that both aim to reduce the burden of chronic diseases and promote healthy eating and sustainable diets [11,90]. By referencing and incorporating the data-derived dietary patterns from this study, obesity prevalence and risk of NCDs may be reduced. For example, promoting more vegetable consumption and less meat consumption in rural areas and to households with minors via targeted advertising and promotions at grocery stores might help people transition to healthier diets.

III. Strengths and limitations

This study had several strengths; the sampling method for this study was deemed effective for successfully realising research objectives, as it facilitated maximal dissemination of the survey’s questionnaire component, ensured standardised questioning, increased privacy and confidentiality of respondents, allowed for electronic data processing, and permitted data collection within a neutral environment [91,92]. Moreover, this study contained a large representative sample size for the ROI, and subsequent statistical analysis revealed novel dietary patterns and showed that PCA-derived dietary patterns may be a better predictor for socioeconomic and health outcomes. Further, the dietary data collated and employed within the current study are significantly more recent (July–December 2021) than previous studies. 

This study employed PCA to investigate dietary patterns within an Irish cohort; however, several other algorithms exist (i.e., hierarchical/two-step agglomerative cluster analyses and latent profile analysis). While the use of PCA is typically more straightforward and logical than cluster analysis [50,56], there are some inherent limitations that should be noted. For example, the user is required to make subjective decisions during the process, such as selecting an appropriate number of components (e.g., the eigenvalue cut-off) and assigning the appropriate cluster to each respondent based on factor loadings and food consumption frequencies [50,56,93]. PCA is an unsupervised learning algorithm that identifies directions of maximum variance regardless of class labels while latent profile analysis (LPA) is a supervised learning algorithm that finds directions of maximum class separability [94]. LPA is capable of classifying individuals into mutually exclusive groups based on food intake that can then estimate the risk of an outcome for a target group [94,95,96]. This research utilised PCA to identify dietary patterns, as PCA is user friendly and provides two main elements, the scores and loadings, which help identify trends in the data [54,95]. Future work might benefit from analysing the same dataset using cluster analysis and LPA. Additionally, it should be noted that only associations, not causation, can be examined when interpreting odds ratios.

As with any survey, memory bias must be considered when designing any questionnaire, and, in particular, questions relating to consumption recall, where long-term memory plays a role [97,98]. Additionally, previous studies have found that respondents may underestimate their weight by approximately 10%, which impacts BMI calculations [12], with physical measurement of respondent weight and height likely yielding more accurate BMI. As this study did not explore nutrition, physical activity, and alcohol consumption, all of which may significantly impact respondent health, caution is advised when interpreting the results. Dietary data were also collected via a short, generalised FFQ and did not consider the consumption of food products such as processed meats, meat substitutes, and ultra-processed foods [69]. Whether BMI variations between the diet groups is predominantly due to their diet or in combination with other lifestyle factors remains challenging to determine [67].

## 5. Conclusions

The present study successfully employed PCA to identify five distinct dietary patterns among a representative sample of 957 adult respondents in the ROI and identified associations with self-reported health outcomes and socioeconomic variables. The five PCA-derived dietary patterns were “meat-focused”, “dairy/ovo-focused”, “vegetable-focused”, “seafood-focused”, and “potato-focused”. Based on the results from the present study, data-derived dietary patterns may be a better predictor for health outcomes than self-reported dietary patterns. Accordingly, promoting dietary change to healthier diets may lead to a reduction in BMI and a subsequent reduction in NCD occurrence.

## Figures and Tables

**Table 1:** Socioeconomic and health characteristics of respondents from the Health, Environmental, and Economic Impact of Diets (HEED) Survey (*n* = 957).

Characteristics	Total (*n* = 957)		Male (*n* = 403)		Female (*n* = 554)	
Gender	***n***	**%**	***n***	**%**	***n***	**%**
Male	403	42.1	-		-	
Female	554	57.9	-		-	
Age range						
18–24 years	139	14.5	58	14.4	81	14.6
25–34 years	310	32.4	121	30.0	189	34.1
35–44 years	279	29.2	104	25.8	174	31.6
45–54 years	138	14.4	64	15.9	74	13.4
55–64 years	62	6.5	34	8.4	28	5.1
65+ years	29	3.0	22	5.5	7	1.3
Ethnicity						
Irish	754	78.8	315	78.2	439	79.2
European/non-Irish	126	13.2	53	13.2	73	13.2
African	17	1.8	11	2.7	6	1.1
Asian	46	4.8	19	4.7	27	4.9
Mix/other	14	1.5	5	1.2	9	1.6
Settlement pattern						
Urban	678	70.8	296	73.4	382	69.0
Rural	279	29.2	107	26.6	172	31.0
Pre-tax household income ^a^						
<EUR 24,999	87	10.0	41	10.9	46	9.2
EUR 25,000–EUR 49,999	266	30.5	110	29.3	156	31.3
EUR 50,000–EUR 74,999	217	24.9	94	25.1	123	24.7
EUR 75,000–EUR 99,999	141	16.2	60	16.0	81	16.3
EUR 100,000–EUR 124,999	92	10.5	39	10.4	53	10.6
EUR 125,000–EUR 149,999	44	5.0	18	4.8	26	5.2
>EUR 150,000	26	3.0	13	3.5	13	2.6
Respondent education ^a^						
Secondary school	149	15.7	68	17.0	81	14.7
Technical or vocational	126	13.2	72	18.0	54	9.8
Undergraduate degree	275	28.9	114	28.6	161	29.2
Postgraduate diploma or degree	345	36.3	125	31.3	220	39.9
Doctorate	56	5.9	20	5.0	36	6.5
Respondent work status ^b^						
Working for payment or profit	658	69.2	285	71.6	373	67.5
Looking for first regular job	18	1.9	12	3.0	6	1.1
Unemployed	27	2.8	12	3.0	15	2.7
Student with a parttime job	76	8.0	24	6.0	52	9.4
Student without a parttime job	69	7.3	29	7.3	40	7.2
Looking after home/family	40	4.2	6	1.5	34	6.1
Retired from employment	23	2.4	14	3.5	9	1.6
Unable to work	14	1.5	8	2.0	6	1.1
Other	26	2.7	8	2.0	18	3.3
Respondent occupation ^b,c^						
Education	134	18.4	40	13.0	94	22.3
Sales, business, law, and commerce	120	16.5	49	16.0	71	16.8
Computing, IT, scientific and technical	104	14.3	64	20.8	40	9.5
Engineering, architecture, manufacturing, building, construction	67	9.2	48	15.6	19	4.5
Farming, fishing, forestry, and veterinary	27	3.7	21	6.8	6	1.4
Healthcare	82	11.2	16	5.2	66	15.6
Social services	12	1.6	1	0.3	11	2.6
Services	112	15.4	43	14.0	69	16.4
Other	71	9.7	25	8.1	46	10.9
Household composition						
Living alone	160	16.7	75	18.6	85	15.3
Living with other adults and/or minors	797	83.3	328	81.4	469	84.7
Living with minors	394	41.2	167	41.4	227	41.0
Living with adults and no minors	563	58.8	236	58.6	327	59.0
Household composition ^d,e^						
Children (<18 years old)	0.77	1.1	0.79	1.1	0.75	1.1
Adults (≥18 years old)	2.29	1.1	2.19	1.0	2.36	1.1
Total household members	3.06	1.6	2.98	1.5	3.12	1.6
**Health and dietary profile**						
BMI class ^f^						
Underweight (BMI < 18.5)	31	3.4	16	4.1	15	2.8
Healthy (BMI 18.5–24.9)	366	39.8	147	37.7	219	41.3
Overweight (BMI 25.0–29.9)	302	32.8	149	38.2	153	28.9
Obese (BMI > 30.0)	221	24.0	78	20.0	143	27.0
Mean BMI (kg/m^2^) ^d,e^	26.99	6.6	26.71	5.9	27.21	7.0
Median BMI (kg/m^2^)	25.89	-	25.00	-	25.73	-
Self-reported health conditions ^g^						
Hypertension	99	10.3	49	12.2	50	9.0
Diabetes	47	4.9	25	6.2	22	4.0
Coronary heart disease	17	1.8	10	2.5	7	1.3
Self-reported dietary pattern						
Omnivore	606	63.3	226	66.0	340	61.4
Flexitarian	218	22.8	93	23.1	125	22.6
Pescatarian	32	3.3	9	2.2	23	4.2
Vegetarian	76	7.9	28	6.9	48	8.7
Vegan	25	2.6	7	1.7	18	3.2
Self-reported duration of current diet						
Less than 1 year	92	9.7	46	11.5	46	8.4
1 to 5 years	236	24.9	105	26.2	131	23.9
6 to 10 years	78	8.2	31	7.7	47	8.6
11 to 15 years	28	3.0	12	3.0	16	2.9
More than 15 years	515	54.3	207	51.6	308	56.2

^a^ Household pre-tax income reported by respondent; ^b^ based on the pre-existing Irish Census framework; ^c^ only respondents who reported “working for payment or profit” or “student with a part-time job”; ^d^ mean values calculated; ^e^ standard deviation; ^f^ BMI classes were grouped based on the calculated individual respondents’ BMI; ^g^ past or present self-reported health conditions.

**Table 2:** Significant relationships between food group consumption and self-reported dietary patterns (omnivore, flexitarian, pescatarian, vegetarian, and vegan).

Food Group	Test Statistic	*p*-Value
Bread, rice, pasta, grains, oats	15.443	0.492
Vegetables	26.415	0.048 *
Potatoes	36.410	0.003 *
Bananas, avocados, citrus fruit	16.616	0.411
Other fruit	15.212	0.509
Nuts and seeds	73.208	<0.001 *
Fruit and vegetable juice	11.962	0.747
Tea	20.468	0.200
Coffee	21.874	0.147
Dairy	291.397	<0.001 *
Eggs	366.496	<0.001 *
Seafood	193.485	<0.001 *
Red meat	603.364	<0.001 *
Non-red meat	622.483	<0.001 *
Confectionary	20.589	0.195

* Denotes significant differences at 0.05 level between self-reported dietary patterns and food group based on chi-square tests.

**Table 3:** Factor loadings for significant food groups within the five principal components identified using varimax rotation.

	Dietary Patterns				
	PC1	PC2	PC3	PC4	PC5
Food group (variance %)	“Meat-focused” (28.7)	“Dairy/ovo-focused” (18.3)	“Vegetable-focused” (12.1)	“Seafood-focused” (10.6)	“Potato-focused” (9.3)
Non-red meat	**0.885**	0.128			
Red meat	**0.786**	0.181		0.213	0.223
Dairy	**0.256**	**0.838**		−0.121	
Seafood	0.209			**0.865**	
Potatoes	0.154				**0.956**
Nuts and seeds	−0.203		**0.655**	**0.446**	
Vegetables			**0.891**		
Eggs		**0.706**		**0.391**	**0.252**

Loadings X ≥ ±0.25 are shown in bold.

**Table 4:** Socioeconomic characteristics for each of the five PCA-derived dietary patterns: “meat-focused”, “dairy/ovo-focused”, “vegetable-focused”, “seafood-focused”, “potato-focused”, and the presence of statistical differences within each variable.

Socio-Economic Characteristics	“Meat-Focused”	“Dairy/Ovo-Focused”	“Vegetable-Focused”	“Seafood-Focused”	“Potato-Focused”	*p*-Value for Target Characteristic
	PC1 (*n* = 296)	PC2 (*n* = 254)	PC3 (*n* = 212)	PC4 (*n* = 92)	PC5 (*n* = 103)	
	*n* (%)	*n* (%)	*n* (%)	*n* (%)	*n* (%)	
Sex						<0.001 *
Male	135 (45.6)	108 (42.5)	63 (29.7)	45 (48.9)	52 (50.5)	
Female	161 (54.4)	146 (57.5)	149 (70.3)	47 (51.1)	51 (49.5)	
Age group						0.067
18–24 years	46 (15.5)	41 (16.1)	31 (14.6)	11 (12.0)	10 (9.7)	
25–34 years	105 (35.5)	81 (31.9)	71 (33.5)	31 (33.7)	22 (21.4)	
35–44 years	83 (28.0)	73 (28.7)	59 (27.8)	27 (29.3)	37 (35.9)	
45–54 years	38 (12.8)	41 (16.1)	23 (10.8)	13 (14.1)	23 (22.3)	
55–64 years	14 (4.7)	15 (5.9)	20 (9.4)	4 (4.3)	9 (8.7)	
65+ years	10 (3.4)	3 (1.2)	8 (3.8)	6 (6.5)	2 (1.9)	
Ethnicity						<0.001 *
Irish	248 (83.8)	194 (76.4)	153 (72.2)	64 (69.6)	95 (92.2)	
European/non-Irish	25 (8.4)	35 (13.8)	45 (21.2)	18 (19.6)	3 (2.9)	
African	9 (3.0)	3 (1.2)	0 (0.0)	2 (2.2)	3 (2.9)	
Asian	11 (3.7)	19 (7.5)	11 (5.2)	4 (4.3)	1 (1.0)	
Mix/other	3 (1.0)	3 (1.2)	3 (1.4)	4 (4.3)	1 (1.0)	
Settlement pattern						<0.001 *
Urban	186 (62.8)	189 (74.4)	172 (81.1)	74 (80.4)	57 (55.3)	
Rural	110 (37.2)	65 (25.6)	40 (18.9)	18 (19.6)	46 (44.7)	
Pre-tax household income ^a^						0.559
<EUR 24,999	27 (9.1)	20 (7.9)	24 (11.3)	10 (10.9)	6 (5.8)	
EUR 25,000–EUR 49,999	78 (26.4)	68 (26.8)	61 (28.8)	26 (28.3)	33 (32.0)	
EUR 50,000–EUR 74,999	81 (27.4)	55 (21.7)	40 (18.9)	20 (21.7)	21 (20.4)	
EUR 75,000–EUR 99,999	45 (15.2)	37 (14.6)	34 (16.0)	8 (8.7)	17 (16.5)	
EUR 100,000–EUR 124,999	23 (7.8)	26 (10.2)	19 (9.0)	11 (12.0)	13 (12.6)	
EUR 125,000–EUR 149,999	10 (3.4)	17 (6.7)	8 (3.8)	5 (5.4)	4 (3.9)	
>EUR 150,000	10 (3.4)	10 (3.9)	3 (1.4)	1 (1.1)	2 (1.9)	
Respondent education ^b^						0.073
Secondary school	46 (15.5)	41 (16.1)	27 (12.7)	13 (14.1)	22 (21.4)	
Technical or vocational	46 (15.5)	27 (10.6)	21 (9.9)	13 (14.1)	19 (18.4)	
Undergraduate degree	93 (31.4)	71 (28.0)	56 (26.4)	24 (26.1)	31 (30.1)	
Postgraduate diploma or degree	97 (32.8)	99 (39.0)	89 (42.0)	32 (34.8)	28 (27.2)	
Doctorate	13 (4.4)	15 (5.9)	18 (8.5)	8 (8.7)	2 (1.9)	
Respondent employment status ^b^						0.023 *
Working for payment or profit	205 (69.3)	185 (72.8)	133 (62.7)	60 (65.2)	75 (72.8)	
Looking for first regular job	8 (2.7)	3 (1.2)	2 (0.9)	2 (2.2)	3 (2.9)	
Unemployed	6 (2.0)	5 (2.0)	10 (4.7)	2 (2.2)	4 (3.9)	
Student with a parttime job	22 (7.4)	22 (8.7)	20 (9.4)	9 (9.8)	3 (2.9)	
Student without a parttime job	23 (7.8)	19 (7.5)	20 (9.4)	5 (5.4)	2 (1.9)	
Looking after home/family	10 (3.4)	14 (5.5)	5 (2.4)	3 (3.3)	8 (7.8)	
Retired from employment	7 (2.4)	1 (0.4)	8 (3.8)	4 (4.3)	3 (2.9)	
Unable to work	6 (2.0)	3 (1.2)	2 (0.9)	1 (1.1)	2 (1.9)	
Other	8 (2.7)	1 (0.4)	12 (5.7)	4 (4.3)	1 (1.0)	
Respondent occupation ^b,c^						0.017 *
Education	32 (10.8)	37 (14.6)	43 (20.3)	12 (13.0)	10 (9.7)	
Sales, business, law, and commerce	44 (14.9)	39 (15.4)	19 (9.0)	6 (6.5)	12 (11.7)	
Computing, IT, scientific and technical	29 (9.8)	30 (11.8)	21 (9.9)	11 (12.0)	13 (12.6)	
Engineering, architecture, manufacturing, building, construction	30 (10.1)	12 (4.7)	8 (3.8)	11 (12.0)	6 (5.8)	
Farming, fishing, forestry, and veterinary	8 (2.7)	11 (4.3)	2 (0.9)	1 (1.1)	5 (4.9)	
Healthcare	21 (7.1)	27 (10.6)	16 (7.5)	10 (10.9)	8 (7.8)	
Social services	4 (1.4)	3 (1.2)	2 (0.9)	1 (1.1)	2 (1.9)	
Services	38 (12.8)	30 (11.8)	17 (8.0)	10 (10.9)	17 (16.5)	
Other	18 (6.1)	18 (7.1)	23 (10.8)	7 (7.6)	5 (4.9)	
Household composition						
Living alone	48 (16.2)	38 (15.0)	39 (18.4)	23 (25.0)	12 (11.7)	0.113
Living with other adults and/or minors	248 (83.8)	216 (85.0)	173 (81.6)	69 (75.0)	91 (88.3)	0.113
Living with minors	120 (40.5)	114 (44.9)	66 (31.1)	37 (40.2)	57 (55.3)	<0.001 *
Living with adults and no minors	128 (43.2)	104 (40.9)	107 (50.5)	32 (34.8)	34 (33.0)	0.018 *
Household composition ^d,e^						
Children (<18 years old)	0.74 (1.1)	0.92 (1.2)	0.53 (0.9)	0.68 (1.0)	1.06 (1.1)	<0.001 *
Adults (≥18 years old)	2.33 (1.1)	2.30 (1.1)	2.34 (1.1)	2.07 (1.1)	2.26 (1.0)	0.168
Total household members	3.06 (1.6)	3.22 (1.6)	2.87 (1.4)	2.75 (1.6)	3.32 (1.5)	0.005 *
Monthly household food expenses (EUR/month)						<0.001 *
Mean (SD) Median	679 (644.2) 500	819 (718.1) 600	623 (554.8) 450	590 (525.1) 400	798 (639.4) 600	
Duration of current diet						<0.001 *
Less than 1 year	30 (10.3)	25 (9.9)	15 (7.1)	16 (17.6)	6 (5.9)	
1 to 5 years	38 (13.0)	64 (25.3)	83 (39.2)	31 (34.1)	20 (19.8)	
6 to 10 years	15 (5.1)	20 (7.9)	27 (12.7)	7 (7.7)	9 (8.9)	
11 to 15 years	4 (1.4)	11 (4.3)	10 (4.7)	3 (3.3)	0 (0)	
More than 15 years	205 (70.2)	133 (52.6)	77 (36.3)	34 (37.4)	66 (65.3)	

* Denotes significant differences at 0.05 level between the PCA-derived dietary patterns. ^a^ Household pre-tax income reported by respondent; ^b^ based on the pre-existing Irish Census framework; ^c^ only respondents who reported “working for payment or profit” or “student with a part-time job”; ^d^ mean values calculated; ^e^ standard deviation

**Table 5:** Adjusted odds ratio (aOR), and confidence interval (CI) for significantly associated PCA-derived dietary pattern and socioeconomic profiles, arranged from most likely to least likely, based on post-hoc analysis.

PCA-Derived Diet	Socioeconomic Variable	aOR	CI
Meat-focused	Diet duration: more than 15 years	2.64	[1.97, 3.54]
	Occupation: engineering, architecture, manufacturing, building, construction	1.96	[1.18, 3.26]
	Settlement pattern: rural	1.72	[1.28, 2.31]
	Education: postgraduate qualification	0.75	[0.57, 0.996]
	Ethnicity: European/non-Irish	0.51	[0.32, 0.81]
	Diet duration: 6 to 10 years	0.51	[0.29, 0.91]
	Diet duration: 1 to 5 years	0.35	[0.24, 0.51]
Dairy/ovo-focused	Ethnicity: Asian	2.02	[1.11, 3.71]
	Employment status: Retired	0.12	[0.02, 0.91]
Vegetable-focused	Diet duration: 1 to 5 years	2.46	[1.77, 3.41]
	Ethnicity: European/non-Irish	2.21	[1.48, 3.30]
	Occupation: education	2.13	[1.40, 3.24]
	Settlement pattern: urban	2.03	[1.39, 2.96]
	Gender: female	1.99	[1.43, 2.76]
	Diet duration: 6 to 10 years	1.96	[1.20, 3.22]
	Education: postgraduate qualification	1.56	[1.15, 2.12]
	Household composition: living with adults and no minors	1.53	[1.13, 2.08]
	Ethnicity: African	0.77	[0.75, 0.80]
	Education: up to and including secondary school	0.67	[0.47, 0.96]
	Household composition: living with minors	0.58	[0.42, 0.80]
	Gender: male	0.50	[0.36, 0.70]
	Settlement pattern: rural	0.49	[0.34, 0.72]
	Diet duration: more than 15 years	0.39	[0.28, 0.53]
Seafood-focused	Ethnicity: Mix/other	3.89	[1.19, 12.65]
	Diet duration: Less than 1 year	2.20	[1.22, 3.96]
	Occupation: Engineering, architecture, manufacturing, building, construction	2.05	[1.02, 4.12]
	Settlement pattern: Rural	0.56	[0.33, 0.96]
	Diet duration: More than 15 years	0.47	[2.99, 0.73]
Potato-focused	Ethnicity: Irish (SR = 1.5)	3.51	[1.68, 7.36]
	Settlement pattern: rural	2.15	[1.42, 3.26]
	Household composition: living with minors	1.90	[1.26, 2.87]
	Education: up to and including secondary school	1.75	[1.15, 2.67]
	Diet duration: more than 15 years	1.68	[1.09, 2.58]
	Education: postgraduate qualification	0.54	[0.34, 0.84]
	Employment status: student without parttime job	0.24	[0.06, 0.98]
	Ethnicity: European/non-Irish	0.18	[0.06, 0.57]

**Table 6:** Health profiles (calculated BMI, BMI class, and health conditions) for each self-reported dietary pattern.

**Self-Reported Dietary Pattern**	**Omnivorous** **(*n* = 606)**	**Flexitarian** **(*n* = 218)**	**Pescatarian** **(*n* = 32)**	**Vegetarian** **(*n* = 76)**	**Vegan** **(*n* = 25)**	***p*-Value**
	*n* (%)	*n* (%)	*n* (%)	*n* (%)	*n* (%)	
Calculated BMI in kg/m^2^						<0.001 *
Mean BMI (SD)	27.40 (6.8)	27.32 (6.7)	24.37 (3.6)	24.89 (5.0)	24.17 (3.3)	
Median BMI	26.15	26.58	23.43	24.81	24.02	
BMI classes ^a^ (%) ^b^						0.005 *
Underweight (BMI < 18.5)	19 (3.2)	6 (2.9)	1 (3.4)	5 (6.8)	0 (0)	
Healthy (BMI 18.5–24.9)	224 (38.3)	75 (35.9)	17 (58.6)	36 (48.6)	14 (60.9)	
Overweight (BMI 25.0–29.9)	181 (30.9)	80 (38.3)	8 (27.6)	25 (33.8)	8 (34.8)	
Obese (BMI > 30.0)	161 (27.5)	48 (23.0)	3 (10.3)	8 (10.8)	1 (4.3)	
Health conditions (%) ^b^						
Hypertension	68 (11.2)	23 (10.6)	2 (6.3)	6 (7.9)	0 (0)	0.347
Diabetes	28 (4.6)	13 (6.0)	2 (6.3)	3 (3.9)	1 (4.0)	0.918
Coronary heart disease	10 (1.7)	5 (2.3)	2 (6.3)	0 (0)	0 (0)	0.208
**PCA-derived dietary pattern**	**“Meat-focused”**	**“Dairy/ovo-focused”**	**“Vegetable-focused”**	**“Seafood-focused”**	**“Potato-focused”**	***p*-value**
	**(*n* = 296)**	**(*n* = 254)**	**(*n* = 212)**	**(*n* = 92)**	**(*n* = 103)**	
	*n* (%)	*n* (%)	*n* (%)	*n* (%)	*n* (%)	
Calculated BMI in kg/m^2^						<0.001 *
Mean BMI (SD)	27.70 (6.58)	27.17 (6.71)	25.83 (5.95)	26.45 (7.43)	27.44 (6.33)	
Median BMI	26.26	26.19	24.68	25.25	26.88	
BMI classes ^a^ (%) ^b^						<0.001 *
Underweight (BMI < 18.5)	6 (2.0)	10 (3.9)	3 (1.4)	7 (7.6)	5 (4.9)	
Healthy (BMI 18.5–24.9)	110 (37.2)	89 (35.0)	106 (50.0)	34 (37.0)	27 (26.2)	
Overweight (BMI 25.0–29.9)	88 (29.7)	88 (34.6)	61 (28.8)	28 (30.4)	37 (35.9)	
Obese (BMI > 30.0)	83 (28.0)	61 (24.0)	34 (16.0)	16 (17.4)	27 (26.2)	
Health conditions (%) ^b^						
Hypertension	24 (8.1)	33 (13.0)	16 (7.5)	12 (13.0)	14 (13.6)	0.126
Diabetes	10 (3.4)	13 (5.1)	13 (6.1)	5 (5.4)	6 (5.8)	0.658
Coronary heart disease	1 (0.3)	5 (2.0)	3 (1.4)	6 (6.5)	2 (1.9)	0.004 *

^a^ BMI classes were grouped based on the calculated individual respondents’ BMI; ^b^ calculated percentage; * denotes significant differences at 0.05 level between the PCA-derived dietary patterns.

**Table 7:** Associations between PCA-derived and self-described dietary patterns and health outcomes among 957 adults to an online survey in the Republic of Ireland.

**PCA-Derived Diet**	**Health Variable**	**aOR**	**CI**
Meat-focused	Obese BMI	1.46	[1.06, 2.00]
Seafood-focused	Coronary heart disease	5.42	[1.96, 15.01]
	Underweight BMI	3.03	[1.27, 7.26]
Vegetable-focused	Healthy BMI	1.90	[1.39, 2.60]
	Obese BMI	0.57	[0.38, 0.85]
Potato-focused	Healthy BMI	0.56	[0.35, 0.89]
**Self-reported diet**	**Health variable**	**aOR**	**CI**
Omnivorous	Obese BMI	1.76	[1.26, 2.44]
Pescatarian	Healthy BMI	2.20	[1.04, 4.66]
Vegetarian	Obese BMI	0.36	[0.17, 0.76]
Vegan	Healthy BMI	2.41	[1.03, 5.62]

## Data Availability

The data presented in this study are available on request from the corresponding author pending reasonable request and submission of research ethics approval to the TU Dublin Research Ethics Committee. The data are not publicly available for ethical/privacy reasons.

## Appendix A

**Table A1:** Questions relating to the individual-level demographic and socioeconomic status of the respondent and their household.

Question	Possible Responses			
Which sex/gender do you identify as?	• Male		• Other	
	• Female		• Prefer not to answer	
What is your current age?	• Under 18		• 45–54	
	• 18–24		• 55–64	
	• 25–34		• 65–74	
	• 35–44		• 75 years or older	
How would you best describe yourself?	• White		• A mix of two or more	
	• Black or Black Irish		• Other	
	• Asian or Asian Irish		• Prefer not to answer	
	• Arab or Arab Irish			
Which of the following best describes your ethnicity?	• Irish		• Japanese	
	• Eastern European		• Indian, Pakistani, or Bangladeshi	
	• Irish Traveller		• Other Asian backgrounds	
	• Other White background		• Middle Eastern	
	• African		• A mix of two or more	
	• Other Black background		• Other	
	• Chinese		• Prefer not to answer	
Including yourself, how many people currently live in your household?	• People 18 years or older: _____			
	• Children and adolescents aged 17 or younger: _____			
What would you estimate your total pretaxed household income?	• Less than EUR 24,999		• Between EUR 125,000 and EUR 149,999	
	• Between EUR 25,000 and EUR 49,999		• More than EUR 150,000	
	• Between EUR 50,000 and EUR 74,999		• I don’t know	
	• Between EUR 75,000 and EUR 99,999		• Prefer not to answer	
	• Between EUR 100,000 and EUR 124,999			
What is your highest level of education to date?	• Secondary school			
	• Technical, vocational, advance certificate, or completed apprenticeship			
	• Undergraduate degree			
	• Postgraduate diploma or degree (postgraduate diploma, masters)			
	• Doctorate (PhD or higher)			
	• Prefer not to answer			
How would you best describe your present employment status?	• Working for payment or profit		• Retired from employment	
	• Looking for first regular job		• Unable to work due to permanent sickness or disability	
	• Unemployed		• Other	
	• Student or pupil with a part-time job		• I don’t know	
	• Student or pupil without a part-time job		• Prefer not to answer	
	• Looking after home/family			
Which of the following best describes your current occupation?	• Education			
	• Sales, business, law, and commerce (including managers, executives, and clerical and office workers)			
	• Computing, IT, scientific and technical			
	• Engineering, architecture, manufacturing, building, construction			
	• Farming, fishing, forestry, and veterinary			
	• Healthcare (including nursing, dental, therapy, rehabilitation, and pharmacy)			
	• Social services (including childcare and youth services, social work and counselling)			
	• Services (including restaurant, retail, Garda Siochána, hotel, catering, sports, transport, security, occupational health and safety, military and defence, and central and local government)			
	• Other			
	• Prefer not to answer			
Which county in Ireland do you currently reside in?	• Antrim	• Galway		• Monaghan
	• Armagh	• Kerry		• Offaly
	• Carlow	• Kildare		• Roscommon
	• Cavan	• Kilkenny		• Sligo
	• Clare	• Laois		• Tipperary
	• Cork	• Leitrim		• Tyrone
	• Derry	• Limerick		• Waterford
	• Donegal	• Longford		• Westmeath
	• Down	• Louth		• Wexford
	• Dublin	• Mayo		• Wicklow
	• Fermanagh	• Meath		• I don’t live in Ireland currently
Do you live within walking distance to the nearest public house, restaurant, or café?	• Yes			
	• No			
	• I don’t know			
How long do you think it takes to get to the closest pub, restaurant, or café from your home by walking?	• 1 to 5 min (0 to 0.5 km)	• 31 to 45 min (2.6 to 4 km)		
	• 6 to 15 min (0.6 to 1.5 km)	• 46 min to an hour (4.1 to 5 km)		
	• 16 to 30 min (1.6 to 2.5 km)	• More than an hour		
How long do you think it takes to get to the closest pub, restaurant, or café from your home by driving (including public transport)?	• 16 to 30 min (13 to 25 km)	• 46 min to an hour (39 to 50 km)		
	• 31 to 45 min (26 to 38 km)	• More than an hour		
I usually went to the shop by ___.	• Foot	• Public transport		
	• Bike	• Other		
	• Car			
Finally, the shop was about ___ away from where I live.	• Sliding bar corresponding with distance min			
About how much do you think you spent on food (groceries, eating out, and takeaway) last month for yourself?	• EUR 100 to EUR 149	• EUR 300 to EUR 349		
	• EUR 150 to EUR 199	• EUR 350 to 399		
	• EUR 200 to EUR 249	• More than EUR 400		
	• EUR 250 to EUR 299	• I don’t know		

**Table A2:** Questions relating to the respondent’s actual and perceived health metrics and the food consumption habits via a semi-quantitative FFQ.

**Self-Reported Health**	**Possible Responses**			
What is your height in either centimetres or feet? (Please fill in one)	• Centimetres: ____			
	• Inches and feet (for example, 5 foot 8 inches would be 5’8”): ____			
What is your current weight in either kilograms, pounds, or stone? (Please fill in one)	• Kilogrammes: _____			
	• Pounds: _______			
	• Stone: _______			
Have you ever experienced any of the following:	• None of the above		• Diabetes	
	• Food poisoning		• Coronary heart disease	
	• Hypertension			
**Dietary habits**	**Possible responses**			
During the past month, including eating at home and in restaurants or ordering delivery and takeaway, how often did you consume at least one serving size (75~100 g or roughly the size of your fist) of the following foods?				
	At least once every day	Almost every day (3–6 times per week)	Rarely (1 or 2 times a month)	Never or I don’t eat
Bread, rice, pasta, grains, oats				
Vegetables				
Potatoes				
Bananas, avocados, and citrus fruit (such as oranges, grapefruit)				
Other fruit (such as berries, apples, peaches)				
Nuts and seeds				
Fruit and vegetable juices				
Tea (caffeinated, decaffeinated and with or without milk and sugar)				
Coffee (caffeinated, decaffeinated and with or without milk and sugar)				
Continued	At least once every day	Almost every day (3–6 times per week)	Rarely (1 or 2 times a month)	Never or I don’t eat
Dairy, milk (whole, low fat, skimmed and including milk in tea and coffee), cream, cheeses, butter, yoghurt ice cream				
Eggs and food made with eggs				
Fish and seafood (including shellfish and freshwater fish)				
Red meat (such as beef and lamb)				
Non-red meat (such as ham, bacon, pork, and chicken)				
Confectionary and desserts (such as cake, chocolate, and biscuits)				
Which phrase do you think most accurately describes your current personal diet?	• I eat meat, fish, and vegetables (omnivorous)			
	• I eat meat, but I try to limit the amount I eat (flexitarian)			
	• I do not eat meat, but I may or may not consume eggs or dairy (vegetarian)			
	• I do not eat any animal-sourced foods (vegan)			
	• I do not eat meat, but I eat fish and seafood (pescatarian)			
	• Other (please specify)			
How long have you followed this particular diet?	• Less than 1 year			
	• 1 to 5 years			
	• 6 to 10 years			
	• 11 to 15 years			
	• More than 15 years			

**Table A3:** Food consumption habits of the total study sample (*n* = 957) from the Health, Environmentalm, and Economic Impact of Diets (HEED) Survey and the generated components from the PCA and sig between the PC (green/light green shading more frequent and yellow/orange/red shading signifying less frequent).

	Total		Male		Female		Meat-Focused		Dairy/Ovo-Focused		Vegetable-Focused		Seafood-Focused		Potato-Focused	
	(*n* = 957)		(*n* = 403)		(*n* = 554)		(*n* = 296)		(*n* = 254)		(*n* = 212)		(*n* = 92)		(*n* = 103)	
	*n*	%	*n*	%	*n*	%	*n*	%	*n*	%	*n*	%	*n*	%	*n*	%
Self-reported dietary pattern																
Omnivorous	606	63.3	226	66	340	61.4	245	82.8	175	68.9	60	28.3	48	52.2	78	75.7
Flexitarian	218	22.8	93	23.1	125	22.6	42	14.2	69	27.2	55	25.9	31	33.7	21	20.4
Pescatarian	32	3.3	9	2.2	23	4.2	1	0.3	1	0.4	23	10.8	7	7.6	0	0
Vegetarian	76	7.9	28	6.9	48	8.7	7	2.4	8	3.1	52	24.5	5	5.4	4	3.9
Vegan	25	2.6	7	1.7	18	3.2	1	0.3	1	0.4	22	10.4	1	1.1	0	0
Consumption and frequency																
Bread, rice, pasta, grains, oats																
At least once every day	612	64	219	54.5	393	70.9	184	62.2	158	62.2	154	72.6	41	44.6	75	72.8
Almost every day	236	24.7	128	31.8	108	19.5	75	25.3	73	28.7	35	16.5	29	31.5	24	23.3
Sometimes	84	8.8	42	10.4	42	7.6	31	10.5	18	7.1	16	7.5	15	16.3	4	3.9
Rarely	19	2	9	2.2	10	1.8	5	1.7	4	1.6	5	2.4	5	5.4	0	0
Never or I don’t eat	5	0.5	4	1	1	0.2	0	0	1	0.4	2	0.9	2	2.2	0	0
Vegetables																
At least once everyday	582	60.9	206	51.2	376	67.9	147	49.7	163	64.2	163	76.9	47	51.1	62	60.2
Almost every day	245	25.6	117	29.1	128	23.1	86	29.1	60	23.6	40	18.9	31	33.7	28	27.2
Sometimes	99	10.4	61	15.2	38	6.9	46	15.5	23	9.1	7	3.3	12	13	11	10.7
Rarely	25	2.6	14	3.5	11	2	12	4.1	8	3.1	1	0.5	2	2.2	2	1.9
Never or I don’t eat	5	0.5	4	1	1	0.2	5	1.7	0	0	0	0	0	0	0	0
Potatoes																
At least once every day	159	16.6	68	16.9	91	16.5	41	13.9	35	13.8	23	10.8	15	16.3	45	43.7
Almost every day	300	31.4	134	33.3	166	30	86	29.1	81	31.9	54	25.5	21	22.8	58	56.3
Sometimes	387	40.5	151	37.5	236	42.7	144	48.6	108	42.5	96	45.3	39	42.4	0	0
Rarely	100	10.5	44	10.9	56	10.1	25	8.4	25	9.8	35	16.5	15	16.3	0	0
Never or I don’t eat	10	1	6	1.5	5	0.7	0	0	4	1.6	4	1.9	2	2.2	0	0
Bananas, avocados, citrus fruit																
At least once every day	284	29.7	113	28	171	30.9	62	20.9	90	35.4	71	33.5	31	33.7	30	29.1
Almost every day	240	25.1	108	26.8	132	23.9	64	21.6	71	28	53	25	25	27.2	27	26.2
Sometimes	296	31	117	29	179	32.4	109	36.8	63	24.8	64	30.2	29	31.5	31	30.1
Rarely	117	12.2	55	13.6	62	11.2	49	16.6	26	10.2	20	9.4	7	7.6	15	14.6
Never or I don’t eat	19	2	10	2.5	9	1.6	12	4.1	4	1.6	3	1.4	0	0	0	0
Other fruit																
At least once every day	293	30.7	107	26.6	186	33.6	53	17.9	89	35	87	41	34	37	30	29.1
Almost every day	298	31.2	124	30.8	174	31.5	87	29.4	95	37.4	58	27.4	32	34.8	26	25.2
Sometimes	243	25.4	114	28.4	129	23.3	101	34.1	46	18.1	46	21.7	16	17.4	34	33
Rarely	100	10.5	46	11.4	54	9.8	39	13.2	22	8.7	17	8	9	9.8	13	12.6
Never or I don’t eat	21	2.2	11	2.7	10	1.8	16	5.4	2	0.8	2	0.9	1	1.1	0	0
Nuts and seeds																
At least once every day	121	12.7	44	11	77	13.9	1	0.3	38	15	64	30.2	17	18.5	1	1
Almost every day	181	19	76	19	105	19	12	4.1	75	29.5	73	34.4	16	17.4	5	4.9
Sometimes	323	33.9	137	34.2	186	33.7	63	21.3	101	39.8	58	27.4	39	42.4	62	60.2
Rarely	262	27.5	113	28.2	149	27	170	57.4	31	12.2	15	7.1	15	16.3	31	30.1
Never or I don’t eat	66	6.9	31	7.7	35	6.3	50	16.9	8	3.1	1	0.5	3	3.3	4	3.9
Fruit and vegetable juice																
At least once every day	121	12.7	63	15.7	58	10.5	29	9.8	41	16.1	26	12.3	12	13	13	12.6
Almost every day	129	13.6	61	15.2	68	12.4	43	14.5	42	16.5	25	11.8	11	12	8	7.8
Sometimes	215	22.6	113	28.2	102	18.5	73	24.7	46	18.1	38	17.9	29	31.5	29	28.2
Rarely	338	35.5	114	28.4	224	40.7	98	33.1	89	35	88	41.5	28	30.4	35	34
Never or I don’t eat	148	15.6	50	12.5	98	17.8	50	16.9	36	14.2	33	15.6	11	12	18	17.5
Tea																
At least once every day	471	49.4	180	44.8	291	52.7	143	48.3	125	49.2	107	50.5	42	45.7	54	52.4
Almost every day	131	13.7	69	17.2	62	11.2	38	12.8	42	16.5	28	13.2	11	12	12	11.7
Sometimes	118	12.4	55	13.7	63	11.4	37	12.5	32	12.6	27	12.7	9	9.8	13	12.6
Rarely	100	10.5	48	11.9	52	9.4	31	10.5	27	10.6	20	9.4	14	15.2	8	7.8
Never or I don’t eat	134	14	50	12.4	84	15.2	46	15.5	28	11	30	14.2	15	16.3	15	14.6
Coffee																
At least once every day	563	48.4	184	45.7	279	50.5	137	46.3	124	48.8	110	51.9	42	45.7	50	48.5
Almost every day	135	14.1	66	16.4	69	12.5	41	13.9	49	19.3	18	8.5	17	18.5	10	9.7
Sometimes	94	9.8	44	10.9	50	9	21	7.1	23	9.1	28	13.2	11	12	11	10.7
Rarely	71	7.4	35	8.7	36	6.5	29	9.8	11	4.3	17	8	4	4.3	10	9.7
Never or I don’t eat	193	20.2	74	18.4	119	21.5	68	23	46	18.1	39	18.4	18	19.6	22	21.4
Dairy																
At least once every day	614	64.3	261	65.1	353	63.7	210	70.9	188	74	115	54.2	20	21.7	81	78.6
Almost every day	191	20	85	21.2	106	19.1	60	20.3	62	24.4	33	15.6	21	22.8	15	14.6
Sometimes	81	8.5	36	9	45	8.1	20	6.8	4	1.6	32	15.1	20	21.7	5	4.9
Rarely	30	3.1	13	3.2	17	3.1	6	2	0	0	9	4.2	15	16.3	0	0
Never or I don’t eat	39	4.1	6	1.5	33	6	0	0	0	0	23	10.8	14	15.2	2	1.9
Eggs																
At least once every day	159	16.6	72	17.9	87	15.7	45	15.2	81	31.9	8	3.8	16	17.4	9	8.7
Almost every day	325	34	123	30.5	202	36.5	89	30.1	144	56.7	40	18.9	25	27.2	27	26.2
Sometimes	351	36.7	162	40.2	189	34.1	120	40.5	28	11	97	45.8	39	42.4	67	65
Rarely	78	8.2	32	7.9	46	8.3	35	11.8	1	0.4	33	15.6	9	9.8	0	0
Never or I don’t eat	44	4.6	14	3.5	30	5.4	7	2.4	0	0	34	16	3	3.3	0	0
Seafood																
At least once every day	24	2.5	15	3.7	9	1.6	0	0	2	0.8	1	0.5	21	22.8	0	0
Almost every day	118	12.3	62	15.4	56	10.1	20	6.8	46	18.1	14	6.6	38	41.3	0	0
Sometimes	394	41.2	172	42.7	222	40.1	147	49.7	117	46.1	68	32.1	26	28.3	36	35
Rarely	256	26.8	98	24.3	158	28.5	95	32.1	54	21.3	55	25.9	7	7.6	45	43.7
Never or I don’t eat	165	17.2	56	13.9	109	19.7	34	11.5	35	13.8	74	34.9	0	0	22	21.4
Red meat																
At least once every day	50	5.2	34	8.5	16	2.9	22	7.4	14	5.5	2	0.9	7	7.6	5	4.9
Almost every day	228	23.9	122	30.3	106	19.2	109	36.8	59	23.2	13	6.1	11	12	36	35
Sometimes	410	43	163	40.5	247	44.8	139	47	132	52	41	19.3	46	50	52	50.5
Rarely	141	14.8	53	13.2	88	16	15	5.1	49	19.3	49	23.1	18	19.6	10	9.7
Never or I don’t eat	124	13	30	7.5	94	17.1	7	2.4	0	0	107	50.5	10	10.9	0	0
White meat																
At least once every day	78	8.2	42	10.4	36	6.5	32	10.8	20	7.9	10	4.7	10	10.9	6	5.8
Almost every day	360	37.8	151	37.6	209	37.9	161	54.4	92	36.2	36	17	25	27.2	46	44.7
Sometimes	355	37.3	156	38.8	199	36.1	96	32.4	126	49.6	46	21.7	41	44.6	46	44.7
Rarely	54	5.7	27	6.7	27	4.9	7	2.4	12	4.7	27	12.7	5	5.4	3	2.9
Never or I don’t eat	106	11.1	26	6.5	80	14.5	0	0	2	0.8	93	43.9	9	9.8	2	1.9
Confectionary																
At least once every day	188	19.7	62	15.4	126	22.8	66	22.3	44	17.3	42	19.8	13	14.1	23	22.3
Almost every day	303	31.7	114	28.3	189	34.2	95	32.1	79	31.1	68	32.1	27	29.3	34	33
Sometimes	320	33.5	138	34.2	182	33	102	34.5	94	37	58	27.4	35	38	31	30.1
Rarely	120	12.6	73	18.1	47	8.5	25	8.4	31	12.2	37	17.5	14	15.2	13	12.6
Never or I don’t eat	24	2.5	16	4	8	1.4	8	2.7	6	2.4	5	2.4	3	3.3	2	1.9

**Table A4:** Animal product food frequency consumption of respondents delineated by self-reported dietary pattern and PCA-derived dietary pattern.

**Consumption and** **Frequency**	**Omnivorous** **(*n* = 606)**		**Flexitarian** **(*n* = 218)**		**Pescatarian** **(*n* = 32)**		**Vegetarian** **(*n* = 76)**		**Vegan** **(*n* = 25)**	
	***n***	**%**	***n***	**%**	***n***	**%**	***n***	**%**	***n***	**%**
Dairy										
At least once every day	435	71.8	126	58.1	14	43.8	35	46.7	4	16.0
Almost every day	111	18.3	52	24.0	7	21.9	20	26.7	1	4.0
Sometimes	36	5.9	23	10.6	6	18.8	15	20.0	1	4.0
Rarely	14	2.3	9	4.1	2	6.3	2	2.7	3	12.0
Never or I don’t eat	10	1.7	7	3.2	3	9.4	3	4.0	16	64.0
Eggs										
At least once every day	113	18.6	33	15.1	2	6.3	11	14.5	1	4.0
Almost every day	214	35.3	75	34.4	12	37.5	23	30.3	2	8.0
Sometimes	230	38.0	85	39.0	14	43.8	20	26.3	2	8.0
Rarely	38	6.3	21	9.6	3	9.4	14	18.4	-	-
Never or I don’t eat	11	1.8	4	1.8	1	3.1	8	10.5	20	80.0
Seafood										
At least once every day	11	1.8	7	3.2	3	9.4	3	3.9	2	8.0
Almost every day	70	11.6	32	14.7	8	25.0	6	7.9	-	-
Sometimes	286	47.2	77	35.3	15	46.9	15	19.7	1	4.0
Rarely	180	29.7	56	25.7	6	18.8	13	17.1	1	4.0
Never or I don’t eat	59	9.7	46	21.1	-	-	39	51.3	21	84.0
Red meat										
At least once every day	39	6.5	6	2.8	1	3.1	4	5.3	1	4.0
Almost every day	184	30.5	38	17.6	1	3.1	4	5.3	-	-
Sometimes	310	51.3	90	41.7	-	-	9	11.8	1	4.0
Rarely	63	10.4	65	30.1	2	6.3	10	13.2	1	4.0
Never or I don’t eat	8	1.3	17	7.9	28	87.5	49	64.5	22	88.0
Non-red meat										
At least once every day	61	10.1	15	6.9	1	3.1	1	1.3	-	-
Almost every day	280	46.4	66	30.6	1	3.1	10	13.2	3	12.0
Sometimes	238	39.4	106	49.1	1	3.1	9	11.8	1	4.0
Rarely	21	3.5	21	9.7	2	6.3	10	13.2	-	-
Never or I don’t eat	4	0.7	8	3.7	27	84.4	46	60.5	21	84.0
	**Meat-focused** **(*n* = 296)**		**Dairy/ovo-focused** **(*n* = 254)**		**Vegetable-focused** **(*n* = 212)**		**Seafood-focused** **(*n* = 92)**		**Potato-focused** **(*n* = 103)**	
	***n***	**%**	***n***	**%**	***n***	**%**	***n***	**%**	***n***	**%**
Dairy	210	70.9	188	74.0	115	54.2	20	21.7	81	78.6
At least once every day	60	20.3	62	24.4	33	15.6	21	22.8	15	14.6
Almost every day	20	6.8	4	1.6	32	15.1	20	21.7	5	4.9
Sometimes	6	2.0	0	0.0	9	4.2	15	16.3	0	0.0
Rarely	0	0.0	0	0.0	23	10.8	14	15.2	2	1.9
Never or I don’t eat										
Eggs	45	15.2	81	31.9	8	3.8	16	17.4	9	8.7
At least once every day	89	30.1	144	56.7	40	18.9	25	27.2	27	26.2
Almost every day	120	40.5	28	11.0	97	45.8	39	42.4	67	65.0
Sometimes	35	11.8	1	0.4	33	15.6	9	9.8	0	0.0
Rarely	7	2.4	0	0.0	34	16.0	3	3.3	0	0.0
Never or I don’t eat										
Seafood	0	0.0	2	0.8	1	0.5	21	22.8	0	0.0
At least once every day	20	6.8	46	18.1	14	6.6	38	41.3	0	0.0
Almost every day	147	49.7	117	46.1	68	32.1	26	28.3	36	35.0
Sometimes	95	32.1	54	21.3	55	25.9	7	7.6	45	43.7
Rarely	34	11.5	35	13.8	74	34.9	0	0.0	22	21.4
Never or I don’t eat										
Red meat	22	7.4	14	5.5	2	0.9	7	7.6	5	4.9
At least once every day	109	36.8	59	23.2	13	6.1	11	12.0	36	35.0
Almost every day	139	47.0	132	52.0	41	19.3	46	50.0	52	50.5
Sometimes	15	5.1	49	19.3	49	23.1	18	19.6	10	9.7
Rarely	7	2.4	0	0.0	107	50.5	10	10.9	0	0.0
Never or I don’t eat										
Non-red meat	32	10.8	20	7.9	10	4.7	10	10.9	6	5.8
At least once every day	161	54.4	92	36.2	36	17.0	25	27.2	46	44.7
Almost every day	96	32.4	126	49.6	46	21.7	41	44.6	46	44.7
Sometimes	7	2.4	12	4.7	27	12.7	5	5.4	3	2.9
Rarely	0	0.0	2	0.8	93	43.9	9	9.8	2	1.9
Never or I don’t eat	210	70.9	188	74.0	115	54.2	20	21.7	81	78.6
